# Changes in Daily Life Conditions among Korean Older Adults after COVID-19 Outbreak

**DOI:** 10.1093/geroni/igab046.3573

**Published:** 2021-12-17

**Authors:** Jiyoung Lyu, Young Bum Kim, Yeon Ok Lim

**Affiliations:** Hallym University, Chuncheon, Kangwon-do, Republic of Korea

## Abstract

The pandemic of COVID-19 has had a significant impact on peoples’ daily life conditions. Therefore, it is worth understanding changes in daily life conditions among older adults after COVID-19 outbreak. This study was aimed to explore the impact of the pandemic on daily life conditions among Korean older adults. To achieve this aim, an online survey was conducted during April, 2021 to include 184 Korean adults aged 60 years or older nationwide. The results are as follows. First, about 54.9% older Koreans reported that their daily life has stopped since COVID-19. Second, the top 3 increased daily life conditions were spending time at home (77.2%), using internet (60.3%), and shopping online (51.6%). Third, the top 3 decreased daily life conditions were traveling (90.2%), meeting with friends (90.2%), and meeting with family members (80.4%). Fourth, the top 3 daily life conditions with no change were sleeping time (72.3%), eating instant food (58.2%), and household income (53.8%). These findings suggest that several daily life conditions changed after COVID-19 outbreak. Further study is needed to examine the impact of these changes on physical and mental health among older adults.

